# Pandemic Nightmares: COVID-19 Lockdown Associated With Increased Aggression in Female University Students' Dreams

**DOI:** 10.3389/fpsyg.2021.644636

**Published:** 2021-03-05

**Authors:** Erica Kilius, Noor H. Abbas, Leela McKinnon, David R. Samson

**Affiliations:** Sleep and Human Evolution Lab, Department of Anthropology, University of Toronto Mississauga, Mississauga, ON, Canada

**Keywords:** COVID-19, dream content analysis, sleep, dream recall, nightmares, threat simulation

## Abstract

The COVID-19 pandemic and its associated stressors have impacted the daily lives and sleeping patterns of many individuals, including university students. Dreams may provide insight into how the mind processes changing realities; dreams not only allow consolidation of new information, but may give the opportunity to creatively “play out” low-risk, hypothetical threat simulations. While there are studies that analyze dreams in high-stress situations, little is known of how the COVID-19 pandemic has impacted dreams of university students. The aim of this study was to explore how the dream content of students was affected during the university COVID-19 lockdown period (March–July, 2020). Using online survey methods, we analyzed dream recall content (*n* = 71) using the Hall-Van de Castle dream coding system and Fisher's exact tests for sex comparisons. Preliminary results indicate that female students experienced more nightmares as compared to male students. Dream analysis found that, relative to normative American College Student (ACS) samples generated pre-COVID-19, women were more likely to experience aggressive interactions in their dream content, including increased physical aggression. Results indicate that university students did experience changes in dream content due to the pandemic lockdown period, with women disproportionally affected. These findings can aid universities in developing support programs for students by bringing forth an understanding of students' concerns and anxieties as they process the “new normal” of social distancing.

## Introduction

On March 16, 2020, the University of Toronto stopped in-person classes at its three campuses in response to the World Health Organization's classification of COVID-19 as a pandemic (University of Toronto News, 2020). On March 19, all public spaces at the university closed and students were asked to vacate student residences if they were able to travel home (Kalvapalle, 2020). Originally intended to last only 2 weeks, the closure of many campus services continued through the winter semester and into the summer, with all classes delivered online. During this time, social distancing protocols were also enforced in the City of Toronto, leading to the closure of municipal public spaces, including libraries, restaurants, and public recreation and fitness centers (City of Toronto, 2020a). A State of Emergency was declared by the City of Toronto on March 23, 2020, mandating that residents stay home for all but essential reasons (City of Toronto, 2020b). Despite lockdown measures, confirmed cases in Toronto rose from 108 on March 17th to 2,363 on April 13th (Toronto Public Health, 2020a,c). Toronto public health officials released daily updates as the pandemic evolved, leading to uncertainty about safety protocols (Toronto Public Health, 2020b); for instance, masks and face coverings were not required in public spaces until July 7th, by which time there had been over 14,000 cases in Toronto (Toronto Public Health, 2020b). The rapid imposition of the Toronto closures left many students isolated and unprepared, without time to develop financial and social supports. A study by Statistics Canada from April 19th to May 1st, 2020 reported that almost 50% of post-secondary students had lost their jobs, while 68% were extremely concerned about depleting their personal savings (Statistics Canada, 2020). This unprecedented large-scale lockdown presented an opportunity to understand how social isolation and stress may impact dream content.

To date, studies analyzing dreams during the COVID-19 pandemic have largely used data from general populations with diverse demographic characteristics (Altena et al., 2020; Pesonen et al., 2020; Schredl and Bulkeley, 2020). Studies within a population with more demographic homogeneity may highlight the shared experience of pandemic lockdown, as similarities of lifestyle, age, and finances may result in further commonalities within dream content. Additionally, dream content research during the pandemic has used predominately quantitative analysis (Barrett, 2020; Mota et al., 2020; Schredl and Bulkeley, 2020); little attention has been given to qualitative themes to explore individual experiences. The University of Toronto student body is highly diverse, comprised of 21% international students from 168 countries (University of Toronto Office of Planning Budget, 2020). This cultural diversity gives further insight while contextualizing the student dream experience during COVID-19 pandemic.

Recent reports on the effects of lockdown have suggested that university students are experiencing heightened anxiety and worry (Husky et al., 2020), a potential consequence of students' sudden displacement from university campuses, health concerns for themselves and their families, and financial and academic concerns (Statistics Canada, 2020). Because dream recall is often short-lived, and the municipal lockdown ended by early July, the window of opportunity to study the lockdown effect on dreams was small. Thus, the primary aim of our study was to examine the dream content of University of Toronto students during the COVID-19 isolation period (March–August, 2020).

Sleep and dreaming during the pandemic have been of much interest to sleep researchers. While the general effect of stress on sleep and dreaming has been thoroughly investigated (Payne and Nadel, 2004; Âkerstedt, 2006), the pandemic's global reach allows comparison of individual responses to a shared external stress that may shape common sleep patterns and dreams. Studies have employed social network analyses to explore pandemic dream content in Finland (Pesonen et al., 2020); dream diaries of university students collected over 2-week periods in Canada (MacKay and DeCicco, 2020), large online surveys in the U.S.A (Schredl and Bulkeley, 2020) and across the globe (Barrett, 2020); and natural language processing methodology to analyze dream reports in Brazil (Mota et al., 2020).

Previous research has demonstrated that nightmares and disrupted sleep increase immediately following a traumatic event (Barrett, 2001; Bulkeley and Kahan, 2008). The mechanism for this phenomenon is uncertain. The dream continuity hypothesis (Domhoff, 2003) posits that the content of dreams is often a reflection of the dreamer's concerns in their waking state. Evolutionary explanations for dreaming also provide context for this phenomenon: Revonsuo (2000) proposed the threat simulation theory (TST), whereby aggressive dreams help an individual practice responding to hypothetical threats in order to strengthen threat response in waking life. Revonsuo and Valli (2008) later clarified that threats are not necessarily physical, but can include what Slavich (2020) calls threats to social safety, including isolation, exclusion from the group, and death or illness of a genetic relative (Revonsuo and Valli, 2008), all of which threaten reproductive success. The TST (Revonsuo, 2000) is supported by the finding that negative events far outweigh positive events in dream content, while in waking life, the opposite is true (Dale et al., 2016). Alternatively, the social simulation theory posits that dreams simulate positive or neutral social interactions to rehearse social activities in waking life (Revonsuo et al., 2015; Tuominen et al., 2019). Regardless, many participants of dream studies believe that dreams can help them make important decisions in waking life, or place value on the information that their dreams provide (Morewedge and Norton, 2009; Salem et al., 2009).

The aim of our project was to better understand how University of Toronto students were interpreting their dreams through the shared experience of university closure and city lockdown. We hypothesized that dream content during the COVID-19 isolation period would differ from pre-pandemic samples; specifically, we predicted pandemic dreams would be characterized by more reports of nightmares, stressful events, and negative emotions. Additionally, we used qualitative methodology to gain insight into the subjective dream experiences of students during COVID-19 lockdown.

## Materials and Methods

### Data Collection and Participants

Our study sample was recruited through posts on university social media pages (Facebook and Reddit) and emails sent to departmental listservs. The survey was conducted using Google Forms (please see Supplementary Material). Participants were University of Toronto students of any age, verified by submitting university-issued email addresses. Survey responses were gathered from July 17th to August 10th, 2020 (24 days). Participants were provided information about the study aims and protocols before beginning the survey, and were informed that their participation was voluntary and could be withdrawn without negative consequences. Consent was indicated by proceeding to the survey. Study protocol followed University of Toronto Human Research Ethics Board protocol #39551.

The survey was divided into three sections. First, participants were asked to “describe a dream you remember having during the COVID-19 isolation period (March 16th–July 25, 2020).” This prompt is similar to the control sample, which asked participants “to describe a dream as they remember it” (Domhoff and Schneider, 1998). Participants were asked to describe their dream in 50–250 words, consistent with the Hall-Van de Castle dream coding requirements (Hall and Van de Castle, 1966). Questions in multiple choice format about the setting and content of the dream followed for further clarification; participants were asked to recall (a) the primary setting of the dream, (b) who was primarily in the dream, (c) whether participants considered the dream to be good, bad, or neutral, and (d) the primary emotions they experienced in this dream. Participants were also asked whether they believed their culture influenced their interpretation of this dream.

The second section surveyed participants about their dreams in general during the COVID-19 isolation period. This included whether participants felt their dreams had changed, and if so, how (in a “check all that apply” format). Additional questions asked whether participants use dreams to make decisions in their everyday lives, and if they had dreamed explicitly about the COVID-19 pandemic (“Yes; No; I Don't Know/I'm Not Sure”). A COVID-19 dream constituted any dream that involved concepts of the pandemic (i.e., dreams about the disease itself, wearing masks and gloves, or social distancing protocols). The final section asked demographic information, including age category, sex, gender, country of origin, self-identified ethnicity, and university department.

### The Hall-Van de Castle Dream Coding System

The Hall-Van de Castle Dream Coding System (HVdC) is an empirical method of dream content analysis, and is the primary method of quantitatively analyzing dream reports (Domhoff and Schneider, 1998; Domhoff, 2003). Dream categories in this study include five categories described by the HVdC system: characters, social interactions (aggressive, friendly, and sexual interactions), use of descriptive elements, settings (physical surroundings), emotions experienced during the dream, and any occurrences of misfortune and good fortune. These categories are generally regarded as the “The Big Five” in the HVdC system and are an often-scored combination when establishing preliminary findings (Domhoff, 2003). Our sample of student dreamers during COVID-19 was then compared to a control normative baseline sample, generated from American College Students (ACS) in a non-pandemic context. The baseline sample consists of student dream reports from Case Western Reserve University, collected between 1947 and 1950 (Domhoff, 2003). In the pre-pandemic sample, five dream reports were collected from 100 females (*n* = 500) and 100 males (*n* = 500).

### Statistical Analysis

General statistics on dreams were compiled in Microsoft Excel and analyzed in R statistical language (R Core Team, 2018). All questions had the options “I don't know” and “Prefer not to answer”; these responses were considered missing data and thus excluded from each relevant question when compiling descriptive results. Four questions in our survey posed questions of magnitude (i.e., if participants were experiencing more or less nightmares during the isolation period; if their dreams were more or less stressful; if they were remembering their dreams more or less often; and if family and friends were in their dreams more or less often). Due to the “select all that apply” option for these questions, only the question regarding nightmares had an effect size large enough for analysis; thus, we compared male and female response frequency of this question with a Fisher's Exact test.

For dream descriptions, scored dream content was input into the Automated Dream Data Entry System and Statistical Analysis Tool (DreamSAT). Due to the method being exclusively functional with percentages, the most appropriate statistical method of determining magnitude of differences of our sample relative to the baseline sample is through the Cohen *h*-statistic (Domhoff, 2003). Statistics concerning effect sizes, namely *h*-scores, of our sample were generated relative to the control group's normative dream data, subcategorized by male or female. This comparative approach identifies sex differences in dream content.

Thematic analysis was employed to identify patterns not captured in the HVdC (1966) coding analysis. The value of qualitative dream analysis is in revealing negative emotions and responses to stress in dream content that may not fall into objective categories (Braun and Clarke, 2006). For this study, we followed Braun and Clarke's (2006) phases for thematic analysis. From the survey responses, dream descriptions were coded for initial themes by E.K. and L.M, who then identified 4 predominant themes: culture-based dreams, COVID-19 specific dreams, school/academic dreams, and general anxiety dreams. E.K., L.M., and N.H.A. then discussed each dream report until consensus was reached about a reports' categorization. Based on the themes above, four dreams were selected that exemplified each theme. A 30-min interview with each participant was conducted to further elucidate dream content and participant interpretation.

## Results

### Demographics

Seventy-one participants completed the survey. Of the survey respondents, 51 identified as women, 19 as men, and 1 non-binary. One dream recollection was not included in the HvDC analysis due to sex-based requirements of the coding system, although their general responses were retained for descriptive statistics. Participant age ranged from 18 to 49, with the majority (*n* = 62) between 18 and 29 years. Self-reported ethnicity comprised 21 White individuals, 16 Chinese, 12 South Asian, 4 East Asian, 2 Filipino, 2 Korean, 1 Indo-Caribbean, 1 Middle East Asian, 1 Arab, 1 Jewish, 1 Black, and 9 selecting more than one ethnicity. Participants represented 22 departments at the University of Toronto; 43 participants were undergraduate, and 27 graduate students (1 preferred not to answer).

### Descriptive Statistics Related to Dreams

Most survey respondents reported that their dreams had changed during the COVID-19 isolation period: 27% reported dreams had changed significantly (*n* = 17 of 63), an 54% (*n* = 34 of 63) reported their dreams had changed a moderate amount (19% reported no change). When asked how their dreams had changed during the pandemic, 55% of participants (*n* = 39 of 71) noted their dreams were more stressful, while 50.7% (*n* = 36 of 71) reported their dreams were more vivid. While 42.2% reported they had experienced more nightmares overall, women were significantly more likely to report an increase in nightmares during the pandemic (Fisher's exact test, OR: 22.31, *p* = 0.006, confidence interval: [1.71–1313.1]). Additionally, over one third of respondents (26 of 65, or 40%), reported they had dreamed specifically about the COVID-19 pandemic.

### Reported Dream Descriptives and Quantitative Analysis

A sample size of 70 dream reports were collected for quantitative dream analysis (51 female dreams and 19 male dreams). In response to questions about their described dream, 47.1% of participants described their dream as a “bad dream” (compared with only 17.1% reporting a good dream; 35.7% reported a neutral dream, *n* = 70). Anxiety (63.4%), confusion (60.6%), and fear (50.7%) were the primary emotions associated with the dream descriptions, although excitement (35.2%) was also commonly reported (*n* = 71). The primary settings of dreams were “a place I did not recognize” (28.2%), although home (19.7%) and school (12.7%) were also reported (*n* = 71). Only 37% of participants (*n* = 17 of 46) believed their culture influenced the dream content.

**Figure 1:**
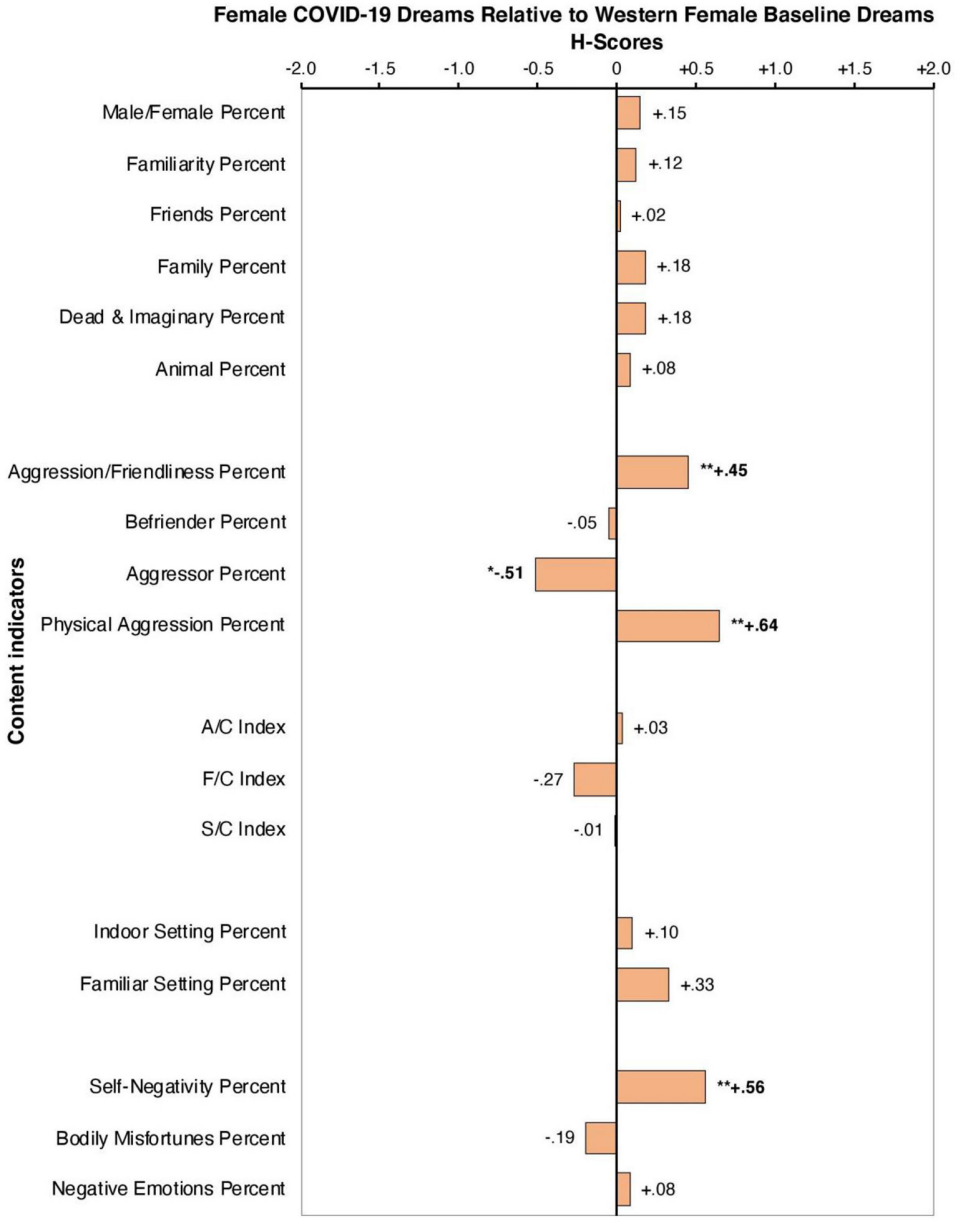
H-profile of female dreamers (*n* = 51) during COVID-19 relative to Western female dreams. *significant at the 0.05 level, **significant at the 0.01 level.

**Figure 2:**
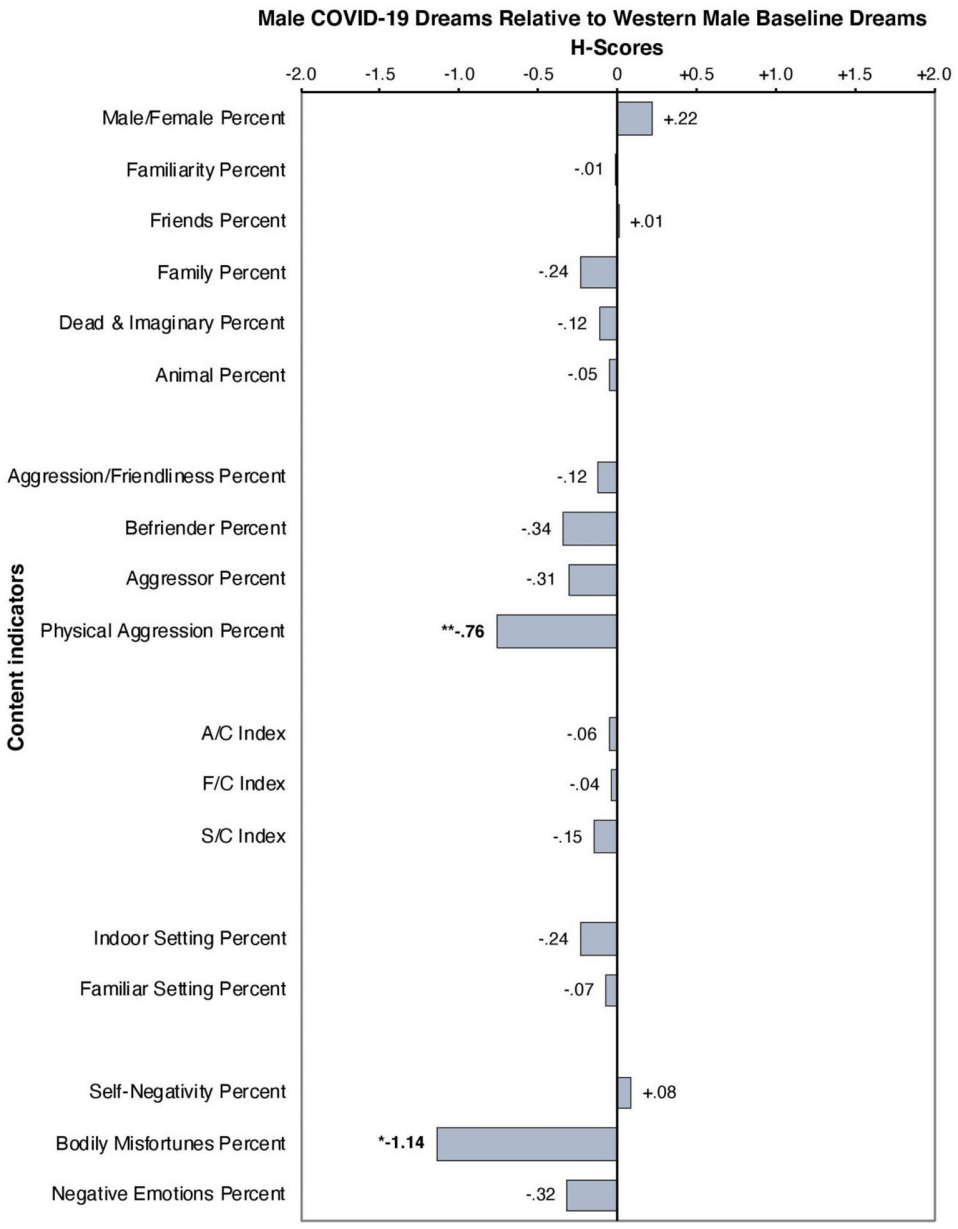
H-profile of male dreamers (*n* = 19) during COVID-19 relative to Western male dreams. *significant at the 0.05 level, **significant at the 0.01 level.

Results of the Hall and Van de Castle (1966) dream coding analysis indicate significant differences in dream content in the context of the pandemic compared to baseline. Relative to the control group, dreams experienced by females (*n* = 51) during COVID-19 were significantly more likely to be aggressive rather than friendly (*h* = +*0.45, p* = *0.008, p* < 0.01), were more likely to be the recipients of aggressive interactions than the initiators (*h* = −*0.51, p* = *0.015, p* < 0.05), and were more likely to experience physical aggression than verbal aggression (*h* = +*0.64*, p = 0.001, p < 0.01). Lastly, female dreamers during COVID-19 were more likely to be involved in overall negative predicaments, known as the self-negativity scale (*h* = +*0.56, p* = *0.000, p* < 0.01) (see Table 1 and Figure 1). This index captures to what extent the dream-self is experiencing failures, misfortunes, and victimization (Domhoff and Schneider, 1998). Additionally, our results indicate that dream content experienced by males (*n* = 19) during COVID-19 was significantly less likely to involve bodily harm, otherwise known as the bodily misfortunes scale (*h* = −*1.14, p* = *0.012, p* < 0.05). In contrast to females, males were significantly less likely to experience physical aggression in dream content relative to baseline measures *(h* = −*0.76, p* = *0.007, p* < 0.01) (See Table 1 and Figure 2).

**Table 1:** Normative content indicator table demonstrating both female (*n* = 51) and male (*n* = 19) dreamers during COVID-19 relative to the control group.

	**Western Females**	**Females during COVID-19**	***h* vs. Western Females:**	***p* vs. Western Females**	**Western Males**	**Males during COVID-19**	***h* vs. Western Males**	***p* vs. Western Males**
**Characters**								
Male/Female %	48%	56%	+0.15	0.327	67%	80%	+0.29	0.263
Familiarity %	58%	64%	+0.12	0.235	45%	45%	−0.00	0.978
Friends %	37%	38%	+0.02	0.874	31%	33%	+0.03	0.875
Family %	19%	27%	+0.18	0.086	12%	05%	−0.25	0.123
Dead and imaginary %	01%	03%	+0.18	0.059	00%	00%	−0.12	0.448
Animal %	04%	06%	+0.08	0.391	06%	05%	−0.07	0.668
**Social interaction percents**								
Aggression/Friendliness %	51%	73%	+0.45	^**^0.008	59%	53%	−0.12	0.595
Befriender %	47%	44%	−0.05	0.875	50%	29%	−0.45	0.244
Aggressor %	33%	12%	−0.51	^*^0.015	40%	22%	−0.38	0.265
Physical aggression %	34%	66%	+0.64	^**^0.001	50%	27%	−0.76	^**^0.007
**Social interaction ratios**								
A/C index	0.24	0.25	+0.03	/	0.34	0.34	+0.00	/
F/C index	0.22	0.10	−0.27	/	0.21	0.20	−0.02	/
S/C index	0.01	0.01	−0.01	/	0.06	0.00	−0.15	/
**Settings**								
Indoor setting %	61%	66%	+0.10	0.488	48%	35%	−0.27	0.228
Familiar setting %	79%	90%	+0.33	0.084	62%	55%	−0.13	0.563
**Self-Concept**								
Self-negativity %	66%	89%	+0.56	^**^0.000	65%	70%	+0.11	0.623
Bodily misfortunes %	35%	26%	−0.19	0.380	29%	00%	−1.14	^*^0.012
Negative emotions %	80%	83%	+0.08	0.458	80%	68%	−0.30	0.089

*Social interaction ratios do not use the h-statistic*.

* *significant at the.05 level*,

** *significant at the 0.01 level. The bold values represent significant p-values*.

## Discussion

### Dream Content

Our hypothesis that more nightmares and stressful events would be reported in dream content was supported. The dreams of University of Toronto students significantly changed during the COVID-19 lockdown period when compared to pre-pandemic normative data. Participants reported experiencing more nightmares and anxiety-based dreams. Additionally, participants reported remembering their dreams more often during the university lockdown period. These findings are consistent with early reports of the effect of the COVID-19 lockdown on sleep: MacKay and DeCicco (2020) reported a higher incidence of location changes and animal imagery in dreams of university students during COVID-19, both of which have been linked to higher anxiety levels in previous studies (King and DeCicco, 2007). Schredl and Bulkeley (2020) reported greater dream recall and a higher likelihood of negative dream content during the pandemic. Pesonen et al. (2020) found that 55% of reported dreams classified as “distressing” were pandemic-specific and included themes such as social distancing, personal protective equipment (PPE), and the infectivity of the virus.

Findings of increased dream recall and more nightmares may be due to increased stress associated with the COVID-19 pandemic, leading to an increase in sleep fragmentation. Previous research has demonstrated that dream recollection increases with frequent awakenings (Brand et al., 2011; van Wyk et al., 2019). In addition, the underlying neural processing of emotional memory consolidation is activated during REM sleep (Scarpelli et al., 2019). Nightmares, with their intensified emotions, are more likely to be remembered. Thus, increased nightmares reported in our results and other studies (MacKay and DeCicco, 2020; Schredl and Bulkeley, 2020) may be indicative of a general decline in sleep quality throughout the pandemic leading to increased dream recall, rather than simply an increase in the proportion of nightmares.

Importantly, we found that themes demonstrating an awareness of the external environment, especially academic, financial, and contagion-based concerns were highly prevalent. COVID-19 safety protocols were commonly reported in dreams. Individuals dreamed of being unable to return home, of PPE, and of personal danger from exposure to the virus while completing common tasks (such as grocery shopping or travel). This can be contextualized by our study occurring in the early months of the pandemic when COVID-19 protocols and guidelines were new and still evolving, leading to uncertainty about best practices. As our results are preliminary, the function of these dreams remains uncertain and depends on individual context. If these stressful dreams mirror daytime activities faced by participants during the pandemic (i.e., if they dreamed of the stressors of grocery shopping nearly as often as they felt those stressors when shopping in daily life), this finding is consistent with the dream continuity hypothesis (Domhoff, 2003). Pesonen et al. (2020) note that these dreams may be a form of memory consolidation in which individuals can integrate new social distancing procedures from their waking lives. Conversely, pandemic-based nightmares may serve as role rehearsal for new, unfamiliar (and potentially dangerous) situations (Revonsuo, 2000). For instance, if stressful dreams involving travel occurred more frequently than the actual chance of the individual traveling during the pandemic, then our findings more align with the threat simulation theory. Thus, data on waking activities are needed in order to further differentiate between the functions of dreams.

### Sex Differences in Dream Aggression

Of particular interest, we found that female participants reported more negative interactions and high levels of aggression in their dream content compared to normative female dream samples. This was evident not only in our dream content coding analysis (Hall and Van de Castle, 1966), but also in theme identification during qualitative analysis. One female participant dreamed she was being chased by a dinosaur-like creature that was destroying the city; her father reassured her they were at a safe distance, “*but I continue[d] running anyways*.” When interviewed, this participant noted that she considered her actions in her dream to be characteristic of her waking life, saying “*I feel like in real life I would have the exact same reaction. I would keep on running just to make sure.”* A second participant dreamed she was in a limestone maze; she had just found the exit when “*something grasped my throat from behind*.” This dream combines two common themes noted in female participants: aggression and violence toward the dreamer, and a theme of being lost in a building and unable to find their way out. In line with the TST (Revonsuo, 2000), these dreams may reflect confusion on how to stay safe during a pandemic one cannot control or change. The repetitive nature of walking through a maze, or non-stop running from a threat, could reflect hypothetical trial-and-error within a dream, practicing threat avoidance skills (Revonsuo, 2000).

Pre-pandemic reports have found that men report more aggression in dreams than women (Nielsen et al., 2003). Our results find the opposite, suggesting that dreams may reflect differences in processing of pandemic stressors between men and women. A growing body of literature reports that women's pandemic dreams significantly vary from the norm compared to men's (Barrett, 2020; Schredl and Bulkeley, 2020)–a finding also reflected in our sample of university students. In a study investigating dream content specific to the COVID-19 pandemic, Barrett (2020) found that for both males and females, dreams of death increased 3-fold over pre-pandemic levels. Female dreams, however, contained increased prevalence of negative emotions including anxiety, sadness, and anger (Barrett, 2020). While gender differences in dream content are reported in normative dream studies (Hall and Van de Castle, 1966; Nielsen et al., 2003; DeCicco, 2007), the consistent findings of heightened negative emotions in women's dreams during the pandemic may be related to women experiencing greater stressors. Barrett (2020) attributed their finding to the fact that women are disproportionally affected by the COVID-19 pandemic. For example, women perform unpaid labor at three times the rate of men, and are most likely to be the caregivers in the family (Barrett, 2020; Power, 2020). The transition to working from home disproportionally burdened women with both tasks (Power, 2020).

The influence of gender roles was noted by participants themselves in our study's dream descriptions. One female participant dreamed they were in a wooden house with no roof, noting “*A family member was ill… It was raining outside. [They were] shivering in the corner*.” The participant noted that perhaps their culture “*makes me feel responsible for the well-being for the elderly in my family. I recall feeling that the whole situation was my fault*.” Additionally, early research has found that during the lockdown, women are more likely to lose their jobs in the pandemic, are more likely than men to have fewer hours of paid wage work (Barrett, 2020; Power, 2020), and experience greater risk of domestic violence (Bradbury-Jones and Isham, 2020). These themes could be reflected in future qualitative dream content analysis.

Viewed through the lens of the threat simulation theory (Revonsuo, 2000), women's dreams as victims of aggression may be rehearsal of their response to the stressors they anticipate in real life. This is consistent with our finding of significant movement on the self-negativity scale in the female sample; females were significantly more likely to be victimized in their dream content, as well as experience failures and misfortunes. Women's dreams may reflect preoccupation with their many threats to social safety, including financial and domestic pressures (Barrett, 2020). Our findings, which complement other studies that have found sex-based differences in dreams during stressful events (Taylor et al., 2008; Barrett, 2020), introduce the intriguing possibility of evolved sex-based responses to external stressors.

Sleep disturbance, including acute insomnia and delayed sleep onset, may serve an evolutionarily adaptive function (Perogamvros et al., 2020). Increased nighttime vigilance in response to threats (such as COVID-19) may be a fear-based survival mechanism, the severity of which depends on individual differences in emotional processing (Perogamvros et al., 2020). The prevalence in our study of increased nightmares experienced by women in response to COVID-19 stressors may have resulted in more fragmented sleep, facilitating nighttime vigilance. Thus, differences in threat and risk processing between men and women reflected in dream content may have evolved to rehearse what Revonsuo (2000) calls threat avoidance programs. This could lead to sex-differentiated behavioral responses to those threats to increase survival. More cross-cultural data are needed to explore this possible evolutionary explanation.

### Limitations and Future Directions

This study has several limitations. First, our survey asked participants to describe any dream that they could remember since the university closed due to COVID-19. This is in contrast to the more common method of asking participants to describe the last dream they remember (Hall and Van de Castle, 1966), and could affect comparability to other studies. Second, our sample size of 71 was below the minimum size of 100 recommended for analysis using the HVdC method. This is particularly the case for male respondents, who were underrepresented in our sample. Our larger female sample size can be explained by women reporting higher dream recall than men (Brand et al., 2011); additionally, women are more likely to participate in survey studies (Tuominen et al., 2019). Therefore, a larger sample is necessary to confirm our findings.

While an initial aim was to assess the contribution of the respondent's culture on their dream interpretation, we found that a large percentage responded that they were unsure if their dreams had any cultural significance (*n* = 25, or 35.2% of responses), or were hesitant to place cultural importance on these dreams. Connections to cultural values were often extrapolated during interviews, outside of the specific dream recall of the survey. Future research should include interviews of all participants to highlight any cultural importance of findings with a larger sample size.

## Conclusion

The findings from our study increase our understanding of how stressful events may be reflected in dreams. Dreams can serve as a form of storytelling, and a way for individuals to express their subjective realities while permitting a degree of separation from their own conscious emotions (Hollan, 2009). Our findings identify emotional themes of anxiety, academic worry, and heightened negative emotions experienced by females that may reflect the most salient concerns intruding on the dreams of university students during the time of university lockdown.

With limited in-person classes re-instated in the Fall 2020 semester, a continuation of this study with objective sleep measurements tracked alongside dream content is ongoing. We aim to compare how sleep and dreaming of University of Toronto students have changed as we adjust to the “new normal” of university life in a pandemic. Additional research into the effects of social isolation on university students is particularly critical, as many universities have entered a second lockdown period due to the pandemic's second wave.

## Data Availability Statement

The raw data supporting the conclusions of this article will be made available by the authors, without undue reservation.

## Ethics Statement

The studies involving human participants were reviewed and approved by Office of Research Ethics at the University of Toronto. The patients/participants provided their written informed consent to participate in this study.

## Author Contributions

EK and LM conceived of the presented idea. EK, NA, and LM performed the data analysis. DS provided feedback throughout the study and helped with interpretation of results. All authors discussed the results and contributed to the final manuscript.

## Conflict of Interest

The authors declare that the research was conducted in the absence of any commercial or financial relationships that could be construed as a potential conflict of interest.
