# Supplementary material for: Pandemic Nightmares: COVID-19 Lockdown Associated With Increased Aggression in Female University Students' Dreams
Source: Front Psychol. 2021 Mar 5;12:644636. doi: 10.3389/fpsyg.2021.644636 (PMC7973031; doi:10.3389/fpsyg.2021.644636)
Supplement: Supplementary file 1 [file Table_1.DOCX]

Supplementary Material

COVID-19 Dream Survey

**PART I: Specific COVID-19 Isolation Period Dream**

We would like you to describe a dream you remember having during the COVID-19 isolation period (March 16^th^, 2020 – present day). **We encourage you to recall this dream prior to reading the following questions.** Please provide as many details as you are able.

1. What was the primary setting of your dream?
   1. Home
   2. School
   3. Workplace
   4. Outdoors
   5. A friend or family member’s house
   6. A city or town in which I am familiar
   7. A place I did not recognize
   8. Other (please specify):
2. Who was in the dream? Were they known to you?
   1. Yes, they were family
   2. Yes, they were friends
   3. Yes, they were acquaintances (e.g., work or school friends)
   4. There were people in my dream, but I did not recognize them
   5. I was alone
   6. Other (please specify):
3. Were there any animals in your dream?
   1. Yes, animals were in my dream
   2. Yes, animals were in my dream, including mythical or unusual creatures
   3. No, there were no animals in my dream
4. Did you experience any emotions in the dream? Check all that apply.
   1. Anger
   2. Disgust
   3. Fear
   4. Anxiety
   5. Confusion
   6. Sadness
   7. Excitement
   8. Happiness
   9. Other (please specify):
5. Do you consider this dream to be a good dream, a bad dream, or a neutral dream?
   1. It was a good dream
   2. It was a bad dream
   3. It was neither good nor bad
6. Please describe the dream as you remember it. Your summary must be between 50 and 250 words.
7. Do you believe that the culture in which you identify influenced this dream?
   1. Yes
   2. No
   3. I don’t know/I’m not sure
8. If yes, how does your culture influence your interpretation of this dream?

**PART II: General Questions**

1. Do you believe that dreams can help you make decisions about your life?
   1. Yes
   2. No
   3. I don’t know/I can’t decide
   4. Prefer not to answer
2. Do you believe family, friends, or Elders can communicate with you through dreams?
   1. Yes
   2. No
   3. I don’t know/I can’t decide
   4. Prefer not to answer
3. Do you believe your dreams have changed during the COVID-19 pandemic?
   1. Yes, my dreams have changed a lot
   2. Yes, my dreams have changed a little bit
   3. No, my dreams have not changed
   4. I don’t know/I can’t decide
   5. Prefer not to answer
4. Have you dreamt specifically about the COVID-19 pandemic (the disease itself, social distancing protocols, masks and equipment, worry about your health or the health of a loved one, etc.)?
   1. Yes
   2. No
   3. I don’t know/I can’t decide
   4. Prefer not to answer
5. During the COVID-19 pandemic… [Please select all that apply]
   1. I remember my dreams more often
   2. I remember my dreams less often
   3. My dreams are more vivid
   4. My dreams are more confusing
   5. My dreams are more realistic
   6. My dreams are more fantastical
   7. My dreams are more stressful
   8. My dreams are less stressful
   9. I’ve experienced more nightmares
   10. I’ve experienced less nightmares
   11. Family and friends play a stronger role in my dreams
   12. I don’t see my family or friends in my dreams as much as I used to.

**PART III: Demographic Information**

1. What is your age category?
   1. 18 – 29
   2. 30 – 39
   3. 40 – 49
   4. 50 – 65
   5. I prefer not to answer
2. Please select the option that best describes you:
   1. Woman
   2. Man
   3. Transgender
   4. Gender fluid
   5. Non-binary
   6. Two spirit
   7. Other (please specify):
   8. I prefer not to answer
3. Please select the option that best describes you:
   1. Female
   2. Male
   3. Intersex
   4. Other (please specify):
   5. Prefer not to answer
4. What is your country of origin? Please select one. [drop down menu was provided]
5. Please indicate how you self-identify. This self-identification is not intended as an indication of one’s place of origin, citizenship, language, or culture, and recognizes that there are differences both between and among subgroups of persons of colour. If you are of mixed descent, please indicate this by checking off all that apply, rather than using the “Other” line, unless parts of your self-identification do not appear in this list.
   1. Aboriginal
   2. Arab
   3. Black
   4. Chinese
   5. Filipino
   6. Japanese
   7. Korean
   8. Latin American
   9. South Asian
   10. South East Asian
   11. West Asian
   12. White
   13. Other (please specify):
6. What is your current level of education?
   1. Undergraduate student
   2. Master’s student
   3. PhD student
   4. Post-doctoral student
   5. I am taking courses at the university, but am not enrolled in a degree program
   6. My student status is not captured in these options
   7. Prefer not to answer
7. What is your current Department at the University of Toronto? [fill in the blank]
